# Perinatal mental health in Malaysia: understanding the treatment gap and recommendations for the future

**DOI:** 10.1192/bji.2022.2

**Published:** 2023-02

**Authors:** Shaeraine Raaj, Vijo Verghese, Myelone Tharmaseelan, Richard Duffy, N. K. S. Tharmaseelan N. K. Sinnadorai

**Affiliations:** 1 Specialist Registrar in Psychiatry, Tallaght University Hospital, Dublin, Ireland. Email: shaeraine@hotmail.com; 2 Registrar in Psychiatry, St Loman's Hospital, Mullingar, Ireland; 3 Medical Officer in Psychiatry, Melaka Hospital, Malaysia; 4 Consultant in Perinatal Psychiatry, Rotunda Hospital, Dublin, Ireland; 5 Professor of Obstetrics and Gynaecology, Manipal University College, Melaka, Malaysia

**Keywords:** Perinatal psychiatry, Malaysia mental health, postpartum depression, gap in services, lack of psychiatrist

## Abstract

Maternal mental health problems are widespread worldwide, especially against the backdrop of population growth. There is an increasing prevalence of perinatal mental illness in low- and middle-income countries, and Malaysia is no exception. Despite significant improvements in the Malaysian mental health system over the past decade, there are substantial gaps in the delivery of perinatal health services in Malaysia. This article seeks to give a general overview of perinatal mental health in Malaysia and provide recommendations for the development of Malaysia's perinatal mental health services.

Malaysia is a multicultural country transitioning from a middle-income to a high-income country, and these rapid cultural and lifestyle changes bring increased levels of perceived stress. The population in Malaysia in 2020 was 32.7 million, 15.9 million of whom were females. The male:female ratio nationally was 106:100 and the birth rate was 16 births per 1000 population.^1^

Globally, maternal mental health problems are considered a significant public health challenge. A systematic review in 2018 reported that 10–20% of individuals experience mental illness during their pregnancy or perinatal period.^2^ This is a highly vulnerable period as individuals negotiate emotional, physical and psychosocial changes. These immense changes can lead to mental disorders such as postpartum depression, postpartum psychosis and anxiety disorders.^3^ Perinatal mental illnesses are associated with increased maternal morbidity and mortality.^2,3^ Several legislative and policy reforms have led to accessible mental health services, with growing evidence indicating the need for a perinatal psychiatry service in Malaysia.^4–6^

This review aims to outline the unmet needs of perinatal psychiatry as a subspecialty and provide recommendations for the development of such services.

## Prevalence of perinatal mental illness in Malaysia

The national mental health survey in 2019 illustrated a higher prevalence of psychiatric morbidity among women than men in Malaysia.^7^ A systematic review published in 2018 found that reported rates of postnatal depression in Malaysia ranged from 6.8 to 27.3%.^5^ These findings indicate a significant prevalence of postnatal depression in Malaysia and a need to improve and provide timely services to fulfil the unmet demands of these patients. Additionally, a 2018 Malaysian cohort study reported the prevalence of antepartum anxiety symptoms to be 28.8% (95% CI 25.8–31.8%).^4^ There is no epidemiological study published on the prevalence of postpartum psychosis in Malaysia.

Perinatal mental illness in Malaysia has a complex relationship with stress, both domestic and work-related, tumultuous life events, interpersonal conflicts with spouse and family, the lack of family support, poor social status, inadequate educational attainment, malnutrition and lack of social awareness**.** These combine with increased urbanisation and globalisation, along with associated cultural changes, economic difficulties and insufficient family supports. This is further compounded by the lack of perinatal mental healthcare, which as a subspecialty has yet to evolve in Malaysia.

## General healthcare in Malaysia

Healthcare in Malaysia is divided into two sectors, a government funded, financially constrained public sector and a booming private sector. Public healthcare is paid for by Malaysian citizens through the general taxation of income. Patients pay nominal fees in the heavily subsidised public sector. The government provides quality healthcare to everyone in Malaysia, through clinics and hospitals nationwide. There is still a shortage of quality healthcare centres in remote parts of the country. However, in urban centres, both the public and private hospitals are world-class and provide state-of-the-art healthcare. The healthcare provided is very comprehensive, including both allopathic and traditional medicine.

In Malaysia, general hospitals located in every state capital serve as tertiary referral centres. All these hospitals include on their staff senior obstetricians and gynaecologists, along with senior consultant psychiatrists and senior specialists in all major disciplines. The states too have many district hospitals in all the district capitals. Many of these hospitals also serve as training centres for interns. The remaining district hospitals are served by visiting psychiatrists and other specialists from the state hospitals, on a weekly basis at least.

## Mental healthcare in Malaysia

Malaysian mental health services were reformed and modernised by the Mental Health Act 2001.^8^ Malaysia had 410 registered psychiatrists as of July 2018. Of these, only half are employed in the public sector under the Ministry of Health (MOH), providing specialist services in a community setting.^8^ The proportion of clinical psychologists in Malaysia is 1 per 100 000 population, with 6 mental health nurses per 100 000 population.^9^ Within the MOH framework there are seven established and recognised subspecialties in psychiatry, i.e. addiction, liaison psychiatry, neuropsychiatry, forensic psychiatry, geriatric psychiatry, community psychiatry, rehabilitation and child psychiatry. However, perinatal psychiatry is currently not recognised as a subspecialty despite the large population^1^ and significant needs.^5^ This may be attributed to the poor distribution of the resources and provisions for mental health in Malaysia. The Ministry of Health had allocated only 1.3% of the total health budget for mental health in 2017 and 2018.^8^ This spending provision is below the World Health Organization (WHO) recommendation,^8^ whereas upper-middle-income countries reported spending an average of 2.4% of their health budget on mental health.

## Healthcare services for individuals of childbearing age

In Malaysia, healthcare services for individuals of childbearing age are delivered by maternal and child health (MCH) clinics.^10^ In rural areas, these healthcare services are managed by community clinics that also provide healthcare for newborns.^10^ These clinics are staffed by family medicine specialists, obstetricians, medical officers, nurse-midwives, physiotherapists, occupational therapists, nurses, assistant medical officers, nutritionists and dietitians.^10^ Primary healthcare personnel are advised to refer patients to the hospitals directly as per established patient-management protocols.^10^ Mental health professionals are not currently part of this multidisciplinary team.

## Mental healthcare services in the perinatal period

In current practice, the nurse-midwife (referred to as ‘bidan’) plays an important role in the community by identifying signs and symptoms of mental illness in antenatal and postnatal mothers.^10^ Individuals are then directly referred from the MCH clinic to the general psychiatry unit for assessment and treatment. Additional training is provided to the nurse or midwife to help them develop a broader understanding of perinatal mental disorders. This training enhances early detection of such disorders and provides basic mental healthcare and treatment for a range of mental health problems in MCH clinics. In Malaysia, obstetricians and family medicine specialists are currently involved in providing antenatal and perinatal mental healthcare and treatment. Consultant obstetricians and family medicine doctors primarily provide psychoeducation and supportive counselling to patients and their families. They do not embark on pharmacotherapy, except for prescribing anxiolytics, until the patient has been reviewed by a psychiatrist. There are no organised regular training schemes for obstetricians on mental health. Thus, there is a need for specialists in this area of expertise to provide ideal and optimum services in perinatal mental healthcare. This highlights the need for the MOH and the universities to revise and bolster the current postgraduate psychiatry curriculum (Masters in Medicine (Psychiatry)) to include perinatal psychiatry on the syllabus (theory and clinical). DSM-5 and ICD-10, although widely adopted in psychiatric settings in Malaysia, are not commonly used in MCH clinics.^10^ Regular workshops, additional training and continuing professional development (CPD) programmes aimed at addressing perinatal mental health issues could be targeted at MCH staff.

## Recommendations for service development

### Immediate strategy

The MOH and the postgraduate training body should seek to strengthen the existing system primarily by increasing training and additional exposure for midwives and medical staff in MCH clinics. This will certainly enhance the detection and treatment of a range of perinatal mental health problems. Home visits are done regularly by midwives during the antenatal and postpartum periods, primarily for obstetric care only. Most midwives are not trained to handle matters and issues related to mental health. Individuals with such problems are referred to the obstetrics department, which would then refer them to a psychiatrist. Broader policy changes may be required to incorporate or make adjustments to social and cultural beliefs in perinatal mental health. This may provide an opportunity for collaboration between the Ministry of Health and the Ministry of Education in terms of planning and delivering perinatal mental health awareness programmes to the general population in Malaysia. There is also a need for epidemiological research studies to better understand the causative factors, the increasing prevalence of perinatal mental illness in Malaysia and the treatment modalities. Additionally, research can help promote and advocate mental health, including increased budget allocation by the government.

### Short-term strategy

There is growing evidence to support an integrated perinatal mental health model programme for pregnant individuals.^11^ This model would include a multidisciplinary team of obstetricians, family medicine specialists, psychiatrists, medical officers, nurse-midwives, physiotherapists, occupational therapists, nurses, assistant medical officers, social workers and nutritionists.^11^ With the development of an integrated perinatal mental health referral pathway, this would assist in early identification and management of perinatal mental health problems during pregnancy and the postnatal period. This would improve the mental healthcare services in Malaysia tremendously. It would be pragmatic to initially focus on the most severe cases but significant impact can only be achieved through preventive and proactive work aimed at addressing a wide range of mental healthcare needs.

### Medium-term strategy

There is a pressing need to increase mental health spending by the MOH to plan, develop and provide a perinatal psychiatry subspecialty in Malaysia. Each maternity hospital/unit must have access to perinatal mental health services to support pregnant individuals with mental health problems during pregnancy and following delivery. Perinatal services could initially evolve with community-based services, through practitioners with an interest and expertise in the area. Broader service-level intervention may be required to screen for and to address psychological birth trauma.

### Long-term strategy

Consideration needs to be given to individuals in the late antenatal and postnatal periods should they be admitted to general psychiatric units. Pregnant patients during these times are at increased risk of developing mental health problems during the perinatal period.^12^ Alternatively, Malaysia could seek to develop mother and baby psychiatric units. These in-patient mental health facilities are specially designed for postpartum individuals with acute severe mental illness during the puerperium.^13^ They should be admitted with their babies. This model of postnatal care is viewed as best practice in countries such as the UK, France and Australia.^13^ A qualitative study described how women experienced frustration at the lack of specialised perinatal support in a general psychiatric ward and struggled to cope with the separation from their baby. They warranted a mother and baby psychiatric unit rather than a general psychiatric ward.^13^

Malaysia has a significant lack of psychiatrists, with just 1.27 per 100 000 population.^8^ This poses a massive challenge to developing and delivering an integrated service model. A study published in 2021 reported that Malaysia needs 3000 psychiatrists to meet the WHO recommendation of 10 psychiatrists per 100 000 population.^8^ This requires collaboration between the MOH and the postgraduate training body to increase the number of postgraduate training posts in psychiatry.^8^ Increasing the number of psychiatrists would facilitate the development of further specialisation, including areas of perinatal and infant mental health, which evidence suggests would reduce maternal and fetal morbidity substantially.^2,3^

## Conclusions

There have been significant reforms to mental healthcare in Malaysia. However, the present public healthcare services in Malaysia do not cater to or provide perinatal mental health services for pregnant mothers. Increased mental healthcare budgetary provisions are needed to address the lack of such services. In the short term, focused training for staff of MCH clinics might deliver significant improvement in mental healthcare. Thus, the need for an increase in the total number of psychiatrists, in addition to enhancing the training of perinatal mental healthcare service providers. This would need training of doctors in perinatal mental healthcare as a subspecialty. It would be an opportune time for the MOH to formulate a programme for training of doctors in this subspecialty.

## Data Availability

Data availability is not applicable to this article as no new data were created or analysed in this study.
